# 

**Published:** 1792

**Authors:** 


### MEDICAL FACTS

AND

### OBSERVATIONS.

---

VOL. II.

